# Evaluation of the usefulness of a D dimer test in combination with clinical pretest probability score in the prediction and exclusion of Venous Thromboembolism by medical residents

**DOI:** 10.1186/s12959-014-0028-7

**Published:** 2014-11-28

**Authors:** Tarek Owaidah, Nahlah AlGhasham, Saad AlGhamdi, Dania AlKhafaji, Bandar ALAmro, Mohamed Zeitouni, Fawaz Skaff, Hazzaa AlZahrani, Adher AlSayed, Naser ElKum, Mahmoud Moawad, Ahmed Nasmi, Mohannad Hawari, Khalid Maghrabi

**Affiliations:** Department of Pathology and Laboratory Medicine, King Faisal Specialist Hospital and Research Center, Riyadh, Saudi Arabia; Department of Pathology, College of medicine, Qassim University, Buraidah, Saudi Arabia; Department of Medicine, King Faisal Specialist Hospital and Research Center, Alfaisal university, Riyadh, Saudi Arabia; Department of Radiology, King Faisal Specialist Hospital and Research Center, Riyadh, Saudi Arabia; King Faisal Cancer Centre, King Faisal Specialist Hospital and Research Center, Riyadh, Saudi Arabia; Department of Biostatistics, Sidra Medical and Research Center, Doha, Qatar; Department of Critical Care Medicine, King Faisal Specialist Hospital and Research Center, Riyadh, Saudi Arabia

**Keywords:** d-dimer, Clinical probability, Deep vein thrombosis, Pulmonary embolism

## Abstract

**Introduction:**

Venous thromboembolism (VTE) requires urgent diagnosis and treatment to avoid related complications. Clinical presentations of VTE are nonspecific and require definitive confirmation by imaging techniques. A clinical pretest probability (PTP) score system helps predict VTE and reduces the need for costly imaging studies. d-dimer (DD) assay has been used to screen patients for VTE and has shown to be specific for VTE. The combined use of PTP and DD assay may improve exclusion of VTE and safely avoid imaging studies.

**Materials and methods:**

We prospectively used the Wells PTP score and a DD test to evaluate 230 consecutive patients who presented with VTE symptoms. The receiver operating characteristic curve was used to identify a new DD cutoff value, which was applied to VTE diagnosis and compared with the upper limit of locally established reference range for prediction of thrombosis alone and in combination with the clinical PTP score.

**Results:**

We evaluated 118 patients with VTE symptoms fulfilling the inclusion criteria, 64 (54.2%) with clinically suspected deep vein thrombosis (DVT) and 54 (45.8%) with symptoms of pulmonary embolism (PE). The PTP was low in 28 (43.8%) and moderate/high in 36 (56.25%) of the suspected DVT patients, and low in 29 (53.7%) and moderate/high in 25 (46.3%) of the suspected PE patients. Eighteen cases were confirmed by imaging studies: 9 DVT and 9 PE. The agreement between confirmed cases and PTP was significant with PE but not DVT. The negative predictive value for both DVT and PE with current DD cutoff value of <250 μg/L DDU was 100%, whereas with the calculated cutoff the NPV was 88%.

**Conclusions:**

We confirm that PTP score is valuable tool for medical residents to improve the detection accuracy of VTE, especially for PE. The DD cutoff value of 250 μg/L FEU is ideal for excluding most cases of low PTP; however, the calculated cutoff was less specific for the exclusion of VTE.

## Introduction

Deep vein thrombosis (DVT) and pulmonary embolism (PE) are common presentations of venous thromboembolism (VTE) that require urgent recognition, diagnosis, and treatment to prevent or minimize the risk of thromboembolic complications and avert the exposure of patients without thrombosis to the risks of anticoagulant therapy and associated morbidity and mortality [1]. Although the clinical symptoms and signs such as dyspnea, pleuritic chest pain, tachypnea, and tachycardia can raise suspicion of PE, and symptoms and signs of swollen, red, tender, and hot lower limbs can raise suspicion of DVT, these are nonspecific and need to be confirmed by further diagnostic and costly imaging techniques [2]. Wells established a clinical prediction rule incorporating signs, symptoms, and risk factors that can accurately be applied to categorize probability for DVT or PE as low, moderate, or high [3]. A recent article suggested that this approach could be further simplified by using only 2 risk categories: DVT unlikely and DVT likely [1]. The Haemostasis and Thrombosis Task Force of the British Committee for Standards in Haematology [4] recommends that to eliminate the need for diagnostic imaging, a combination of d-dimer (DD) assay and pretest probability (PTP) score should give a negative predicative value (NPV) of >98%, which is equivalent to that of compression ultrasonography for proximal DVT [5].

Unfortunately, a lung scan is often nondiagnostic, even when the incidence of PE ranges from 10% to 30%, and necessitates further investigations. The PTP score for PE in low, moderate, and high groups has been reported to be 1.3%, 16.2%, and 37.5%, respectively [6]. The imaging techniques are costly and operator-dependent, with variability in sensitivity that can be <73% in cases of distal DVT [2].

Activation of the coagulation system results in formation of fibrin, which (after being cross-linked by factor XIII) results in a fibrin clot that is subsequently lysed by the activation of the fibrinolytic system, which breaks the fibrin clot to fibrin degradation products. DD is a fibrin-derived fragment that is released into the circulation when cross-linked fibrin is broken down by the fibrinolytic system [7,8]. Recently, the DD assay has gained significance as a tool that helps in clinical decisions about the presence of thrombosis. It is generally accepted that clinical assessment and elevated DD levels have further diagnostic advantages, enabling administration of therapy when imaging is not available [9]. On the other hand, several studies have shown that the DD assay may have a high NPV [9-11].

Different techniques are available for DD level measurement, but many lack precision. Enzyme-linked immunosorbent assay (ELISA) continues to be the gold standard for DD level measurement with high sensitivity, but is time-consuming and lacks specificity [8]. New immunological techniques such as immunoturbidimetry have gained attention because of their high sensitivity (around 98%) with intermediate specificity (better than ELISA) and short process time [12]. The introduction of Point of Care Testing helped further decrease turnaround time; however, poor bioequivalence hampered its use in clinical settings [13]. Both the PTP scoring system and DD assay has resulted in better decision-making and early thrombosis diagnosis [1,14].

In this study, we evaluated the usefulness of PTP scoring system and DD assay as diagnostic tools for front-line clinicians (medical residents) to detect thrombosis in patients presenting with VTE symptoms.

## Materials and methods

### Patients

Over 3 years, a group of senior medical residents prospectively assessed all inpatients and outpatients >18 years of age who presented with suspected PE or DVT for enrollment in this study. Patients were excluded if they had any of the following: 1) a history of venous thrombosis (6 months), 2) recent pelvic surgery (1 month); 3) an indwelling central line; 4) current pregnancy or delivery <6 weeks prior; 5) inability to undergo compression ultrasonography because of physical or technical limitations; 6) contraindication for radiological contrast; 7) a terminal illness with a life expectancy of <3 months; 8) Patients with active cancer; and 9) symptoms that resolved <72 h before presentation. The research committee of King Faisal Specialist Hospital and Research Center, Riyadh, Saudi Arabia had approved this study.

### Clinical evaluation

A senior medical resident evaluated all patients clinically at presentation. A data collection form including exclusion criteria, signs, and symptoms included by Wells PTP for DVT and PE was used (Tables 1 and 2) [1,15]. Regardless of the PTP score, patients underwent the appropriate imaging technique and a sample was collected for DD testing.

**Table 1:** **Clinical model for predicting the pretest probability score of deep vein thrombosis (adapted from Wells et al.** [1]**)**

**Clinical characteristics**	**Score**
Active cancer (patient receiving treatment for cancer within the previous 6 months or currently receiving palliative treatment)	1
Paralysis, paresis, or recent plaster immobilization of the lower extremities	1
Recently bedridden for ≥3 days or major surgery within the previous 12 weeks requiring general or regional anesthesia	1
Localized tenderness along the distribution of the deep venous system	1
Entire leg swollen	1
Calf swelling at least 3 cm larger than that on the asymptomatic side (measured 10 cm below the tibial tuberosity)	1
Pitting edema confined to the symptomatic leg	1
Collateral superficial veins (nonvaricose)	1
Previously documented deep vein thrombosis	1
Alternative diagnosis at least as likely as deep vein thrombosis	−2

A score of ≥2 indicates that the probability of deep vein thrombosis is likely; a score of >2 indicates that the probability of deep vein thrombosis is unlikely. In patients with symptoms in both legs, the leg with more symptoms is used. (Low: 0–1; moderate: 1–2; high: ≥2–3).

**Table 2:** **Rules for predicting the probability of pulmonary embolism (adapted from Wells et al.** [15]**)**

**Variable**	**No. of points**
**Risk factors**	
Clinical signs and symptoms of deep venous thrombosis	3.0
An alternative diagnosis deemed less likely than pulmonary embolism	3.0
Heart rate >100 beats/min	1.5
Immobilization or surgery in the previous 4 weeks	1.5
Previous deep venous thrombosis or pulmonary embolism	1.5
Hemoptysis	1.0
Cancer (receiving treatment, treated in the past 6 months, or palliative care)	1.0
**Clinical probability**	
Low	<2.0
Intermediate	2.0-6.0
High	>6.0

### DD testing

Venous blood was collected by clean venipuncture into 3.2% sodium citrate to a final ratio of 9:1 using Vacutainer tubes (Becton Dickinson, Franklin Lakes, NJ, USA). Each specimen was centrifuged for 10 min at 3000 rpm in a refrigerated centrifuge and plasma was drawn into a clean plastic or polystyrene (12 × 75 mm) tube and stored at 4°C until testing with 24 hr. from collection. DD level was tested using Innovance DD (Dade Behring, Marburg, A Siemens Company, Germany)—which is a latex-enhanced, turbidimetric test based on polystyrene particles covalently linked to a monoclonal antibody (DD5) to the cross-linkage region of cross-linked fibrin degradation products. All samples were tested by BCS Instruments from Siemens (Marburg, Germany). The local reference range was established from 20 normal blood bank donors (25–250 μg/L FEU). All patients had been subjected to radiological studies, Compression ultrasonography for DVT and CT- pulmonary angiography for PE.

### Statistics

Frequency and percentage were used to describe data according to different demographic variables, diagnostic tests, and outcomes for both DVT and PE. Kappa statistic was used to test the agreement between clinical pretest and radiological findings. To obtain the DD cutoff value to rule out DVT and PE, a constructed receiver operating curve (ROC) curve was used considering actual DD results and radiology as the gold standard. The agreement between the new cutoff value and radiology was obtained by the McNemar test. Sensitivity, NPV, specificity, positive predictive value (PPV), and likelihood ratios were calculated on the basis of the diagnostic imaging. P <0.05 was considered significant for all statistical tests. SPSS 20 and Excel 2007 were used for data analysis.

## Results

Of the 230 patients screened during the study enrollment, only 118 fulfilled the inclusion criteria and were included in the analysis. There were 73 (61.9%) women and 45 (38.1%) men with a mean age at presentation of 52.64 years (range, 17–108 years). The main reasons for exclusion were cancer, an indwelling central line, and pregnancy. The overall prevalence of VTE in this cohort of patients was 15.3% (18): 9 DVT and 9 PE.

Based on the PTP score, 64 patients (54.2%) were clinically suspected to have DVT. The most common presentation was a swollen limb in 51 (78.5%) and lower limb pain in 17 (26.2%). Fifty-four (45.8%) were clinically suspected to have PE, with the most common clinical presentations being dyspnea in 42 (77.8%), tachycardia in 14 (25.9%), tachypnea in 14 (25.9%), and pleuritic chest pain in 13 (24.1%). The PTP score of suspected DVT patients (n = 64) was found to be low in 28 (43.8%) and moderate to high in 36 (56.2%), whereas in suspected PE patients (n = 54), it was found to be low in 29 (53.7%) and moderate to high in 25 (46.3%). The incidence of DVT in patients in low and moderate-to-high PTP score was 7.1% and 19.4% respectively, while for PE the incidence was 36% in moderate-to-high PTP score.

The agreement between PTP score and radiology results for VTE in total (DVT &PE) was significant (P <0.01) with sensitivity 88%, specificity 55%, PPV 26%, and NPV 96%. However, sub analysis for DVT and PE showed that it was not significant for suspected DVT (P = 0.160) and significant for suspected PE patients (P <0.01), because all patients with PE who had been confirmed positive by radiology scored moderate-to-high PTP.

We evaluated the diagnostic value of the established upper limit of the local reference range (<250 μg/L FEU) alone against radiology results in patients presenting with symptoms suggestive of DVT or PE. There were 96/118 (81.8%) cases with positive DD but only 18/96 (19%) was confirmed by radiology. The calculated sensitivity, specificity, PPV and NPV was 100%, 22%, 19% and 100% respectively. We assessed the combination of negative DD (<250 μg/L FEU) with PTP score against radiology, 22/118 (18.6%) patients were found not to have VTE regardless of the PTP score (Table 3). This is almost the same as using DD result alone.

**Table 3:** **The combination of the negative current DD cutoff and pretest score compared with radiology**

	**Radiology positive**	**Radiology negative**	**Total**
	**(Disease present)**	**(Disease absent)**	
Test with current cutoff (DD negative with high PTP score)	0	7 (30%)	7 (30%)
Test with current cutoff (DD negative with Low PTP score)	0	15 (70%)	15 (70%)
Total	0	22 (100%)	22 (100%)

**Figure 1:**
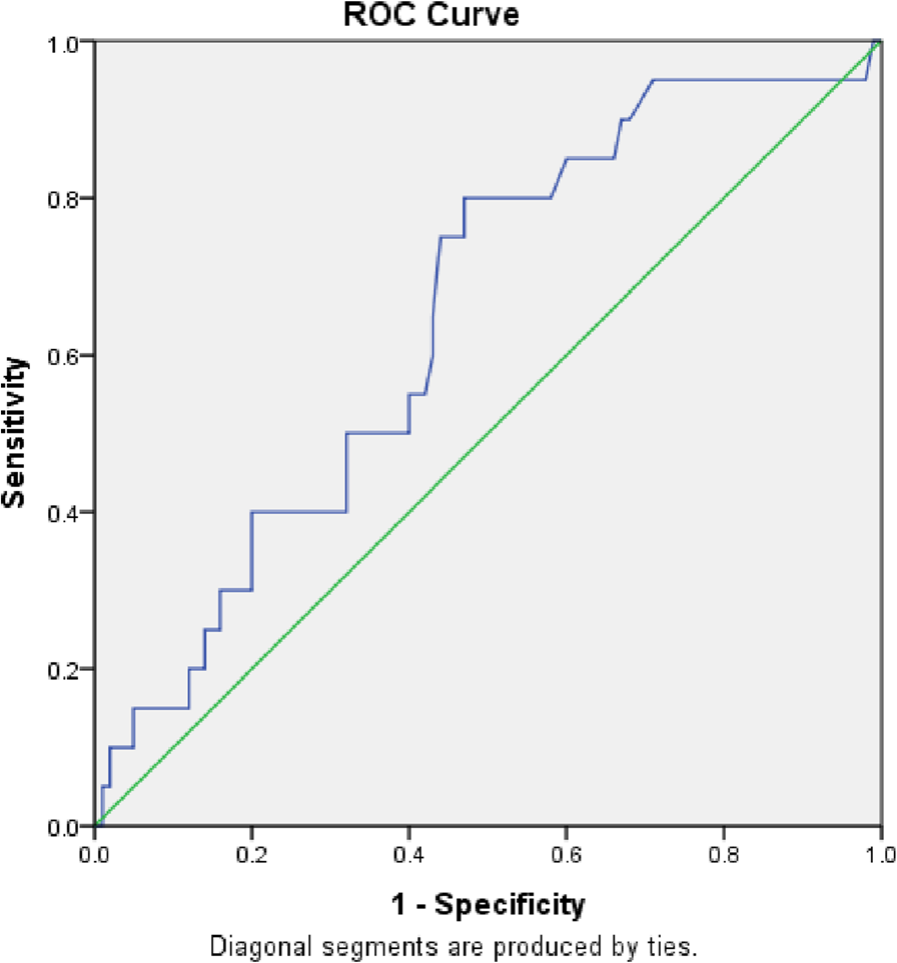
**ROC curve to establish a new cutoff by measured DD and radiology results.**

An ROC curve plotted using the measured DD levels and imaging results was applied to establish a new cutoff value as the point with the highest sum of sensitivity and specificity (60% +63%) for detecting patients with thrombosis (Figure 1). The area under the curve represents the probability that the assay result for a randomly chosen positive case will exceed the result for a randomly chosen negative case. The asymptotic significance is <0.05, which means that results obtained using the assay were better than those obtained without using it.

The calculated DD cutoff value was 815 μg/L FEU for both DVT and PE. To test the accuracy of the calculated cutoff, we used the McNemar test. Considering radiology as the gold standard, we found that the calculated cutoff alone had sensitivity, specificity, PPV and NPV as 88%, 58%, 19%, and 88% respectively. The combination of negative calculated DD cutoff (<815 μg/L FEU) with PTP score identified (56%) 66/118 cases with negative calculated DD in which (88%) 58/66 confirmed negative by radiology and (12%) 8/66 were confirmed positive by radiology (Table 4**)**. These results show that increase in the cutoff of DD showed improvement in PPV with significant reduction in the NPV.

**Table 4:** **Combination between negative calculated DD cutoff and pretest score compared with radiology**

	**Radiology positive**	**Radiology negative**	**Total**
	**(Disease present)**	**(Disease absent)**	
Test with calculated cutoff (DD negative with high PTP score)	8 (12.1%)	25 (37.8%)	33 (50%)
Test with calculated cutoff (DD negative with low PTP score)	0	33 (50%)	33 (50%)
Total	8 (12.1%)	58 (87.8%)	66 (100%)

## Discussion

The evaluation of patients with VTE has been improved by the introduction of standardized probability scoring systems. Different PTP scoring systems have been introduced and evaluated for VTE detection sensitivity and specificity and minimizing the need for radiological confirmation [16-18]. Several studies have investigated PTP score assessment in patients with suspected VTE [19-22]. Wells et al developed a score system calculated from clinical and historical data to stratify patients into low, moderate, and high risk of DVT [3]. Other studies have looked at the significance of the value of these PTP systems in predicting PE [23,24]. The use of DD, the predominant form of a fibrin-degradation product, as a biomarker of thrombosis had been extensively evaluated clinically in the past several decades [11]. DD sensitivity and specificity were the limiting factors for using this test to evaluate thrombosis. Several studies have shown that ELISA is the most sensitive, but with moderate specificity and high NPV; however, it is been hampered by the impracticality of the time needed to perform [8,11]. Many other techniques are available for DD level testing that differ in the principal type of monoclonal antibody specificity that recognizes different epitopes, assay calibration standards, and instrumentation. However, each of these methods has its own limitations and predictive value for detecting or excluding thrombosis [25-28].

We used an immunoturbidimetry (Innovance) assay for measuring DD levels in our hospital. It had been reported to have high sensitivity and NPV but low specificity [5]. De Moerloose et al. evaluated Innovance on different analyzers and found it accurate for the VTE diagnosis workup with a sensitivity >99% and NPV 95.5% [29]. In our study, we found that the sensitivity and specificity depends on the selected cutoff value, whereas the reported current DD cutoff value showed 100% sensitivity and 22% specificity with a NPV of 100% and PPV of 19%. These results were also reported in several studies with an NPV range of 91–96% [25,28,29].

Using a different DD level has been shown to produce a different specificity in excluding VTE. Yamaki et al. showed that the use of ROC curve analysis to select DD cutoff points increased the specificity from 48.9% to 78.2% for the low PTP groups; however, it did not achieve substantial improvement in the moderate- and high-risk PTP groups [30]. We did not find improvement in thrombosis detection by using the ROC curve calculated DD level. Courtney et al. showed the same findings when they tested the different DD levels [23]. Although the sensitivity of most new DD assays, including ours, has been shown to be very high, the specificity is low even in ELISA-based assays [2].

The PTP scoring system proposed by Wells was tested in many studies and was found to be reproducible when used by junior residents [31] and could be used to exclude VTE safely when the score was low [3].

Combination of both the PTP score and DD test value has been suggested as sufficiently accurate for the exclusion of VTE and reduction of the requirement for radiology [9-11,14,32,33]. When we combined both PTP score and DD level to exclude VTE, we identified a low PTP score in 43.1% and moderate to high PTP score in 56.9% of DVT patients with no significant agreement with radiological studies; however, 52.7% and 47.3% of PE patients had a low and moderate-to-high PTP score respectively, with significant agreement of the low PTP score with radiological findings. This can be attributed to more specificity for PE symptoms than for DVT symptoms. Kelly and Hunt found in a pool of data from 4 different studies that 41%, 49%, and 10% of patients had low, moderate, or high PTP score with PE prevalences of 8%, 36%, and 67%, which is not very different from our reported results [7].

In conclusion, according to our data, we are certain that we can rule out thrombosis in patients with negative DD results when combined with low PTP score; however, the PTP score is less sensitive for DVT and cannot rule out patients with thrombosis. There is no need to change the cutoff value because the cut off we used based on reference range of DD level showed very high NPV in VTE and the new cutoff value did not considerably improve the PPV alone or in combination with PTP. Our study has a major limitation, which is its low sample size; however, the results are in agreement with those previously reported.
